# Soy protein concentrate replacing animal protein supplements and its impacts on intestinal immune status, intestinal oxidative stress status, nutrient digestibility, mucosa-associated microbiota, and growth performance of nursery pigs

**DOI:** 10.1093/jas/skac255

**Published:** 2022-08-11

**Authors:** Zixiao Deng, Marcos Elias Duarte, Ki Beom Jang, Sung Woo Kim

**Affiliations:** Department of Animal Science, North Carolina State University, Raleigh, NC 27695, USA; Department of Animal Science, North Carolina State University, Raleigh, NC 27695, USA; Department of Animal Science, North Carolina State University, Raleigh, NC 27695, USA; Department of Animal Science, North Carolina State University, Raleigh, NC 27695, USA

**Keywords:** animal protein, growth performance, mucosa-associated microbiota, nursery pigs, soy protein concentrate

## Abstract

This study was to evaluate the effects of soy protein concentrate (SPC) supplementation replacing animal protein supplements on intestinal immune status, intestinal oxidative stress status, nutrient digestibility, mucosa-associated microbiota, and growth performance of nursery pigs. Thirty-two newly weaned pigs at 21 d of age with 6.4 ± 0.4 kg body weight (BW) were allotted to four treatments in a randomized complete block design with initial BW and sex as blocks. Pigs were fed for 35 d in three phases. Dietary treatments were SPC 0% (diets with fish meal 4/2/1%, poultry meal 10/8/4%, blood plasma 4/2/1%, and crude protein 24.6/22.6/20.9% for phase 1/2/3, respectively), SPC 33%, SPC 66%, and SPC 100% (SPC 0% diets with SPC replacing 33/66/100% of animal protein supplements, respectively). Pigs were euthanized on day 35 to collect jejunal mucosa and tissues to evaluate intestinal immune status, intestinal oxidative stress status, intestinal morphology, and mucosa-associated microbiota in the jejunum. Titanium dioxide was added in phase three diets as an indigestible marker. Ileal digesta was collected to measure apparent ileal digestibility (AID) of nutrients. Data were analyzed using MIXED and NLMIXED procedures of SAS. Increasing SPC supplementation by replacing animal protein supplements linearly decreased (*P* < 0.05) the BW, ADG, and ADFI of pigs during the overall period, and linearly increased (*P* < 0.05) peptide tyrosine tyrosine (PYY) in jejunum. Increasing SPC supplementation linearly decreased (*P* < 0.05) feed cost per weight gain. In the exponential model, SPC can replace animal protein supplements up to 10.5% and 16.5% without reducing the ADG and ADFI of pigs, respectively. The SPC 100% decreased (*P* < 0.05) *Helicobacteraceae*, *Campylobacteraceae*, alpha diversity, and changed beta diversity of microbiota in the jejunal mucosa. In conclusion, SPC supplementation replacing animal protein supplements reduced growth performance by reducing feed intake, which might be related to increased PYY. However, 10.5% and 16.8% of animal protein supplements can be replaced by SPC without affecting BW gain and feed intake of nursery pigs, respectively. Complete removal of animal protein supplements by SPC supplementation modulated the composition of jejunal mucosa-associated microbiota by reducing *Helicobacteraceae* and *Campylobacteraceae*, whereas without affecting the intestinal immune status, intestinal oxidative stress status, intestinal morphology, and AID of nutrients in nursery pigs.

## Teaser

Soy protein concentrate can partly replace animal protein supplements in the diets of nursery pigs without negative growth performance whereas beneficially modulating intestinal microbiota.

## Introduction

Weaning is generally considered the most stressful event for pigs due to the complex changes in the dietary source, physiology, environment, and hierarchy (Lallès et al., 2004; Kim and Duarte, 2021). In addition to the dietary change from sow milk to solid feed, anti-nutritional factors in the feeds can negatively affect the growth and intestinal health of nursery pigs through reduced feed intake (Duarte et al., 2019), increased inflammation (Taliercio and Kim, 2013; Tiwari et al., 2018), and damaged intestinal morphology (Högberg and Lindberg, 2004; Duarte et al., 2021). Collectively, these aforementioned stressors can potentially result in increases of opportunistic harmful bacteria in the jejunal mucosa-associated microbiota of pigs (Duarte and Kim, 2022). Therefore, swine nutritionists have tried various nutritional strategies to minimize the adverse effects of antinutritional factors in the feeds during the post-weaning period.

Soybean meal (SBM), considered as an affordable and high-quality protein supplement, is largely used in swine feeds. However, the antinutritional factors in SBM can impair the growth performance, intestinal morphology, and immune status of nursery pigs (Dunsford et al., 1989; Li et al., 1990). Glycinin and β-conglycinin in SBM can cause allergenic reactions, which stimulate local and systemic immune responses thus negatively affecting the growth performance of nursery pigs (Sun et al., 2008a; Taliercio and Kim, 2014). In addition, due to the lack of endogenous enzymes, the oligosaccharides in SBM such as raffinose and stachyose can also increase digesta viscosity and reduce nutrient digestibility of nursery pigs (Kim et al., 2003; Baker et al., 2010; Pangeni et al., 2017). Therefore, the use of SBM has been partly limited in early nursery feeds.

Animal protein supplements, including blood plasma, fish meal, and poultry meal, have been broadly used in nursery feeds to reduce the negative impacts of weaning stress (Heo et al., 2013). Previous studies have shown that animal protein supplements enhance nutrient digestibility, reduce the inflammatory reaction, and thus improve the growth performance of nursery pigs (Kim and Easter, 2001; Bosi et al., 2004; Keegan et al., 2004). However, there are potential concerns about the use of animal protein supplements in swine feeds due to the affordability, availability, and safety issue (Kim et al., 2019).

Soy protein concentrate (SPC) is produced by removing soluble carbohydrates of defatted flakes to contain fewer oligosaccharides and 15% to 23% higher concentration of crude protein than SBM (Peisker., 2001). Allergenic factors, including glycinin and β-conglycinin, are denatured during the processing under specific ethanol concentration and temperature (Sissons et al., 1982). In addition, previous studies showed that SPC had a higher concentration of DE, ME, and higher digestibility of amino acids than those in SBM (Yang et al., 2007; Zhang et al., 2013; Oliveira and Stein, 2016) providing the rationale for being used to replace animal protein supplements in nursery diets.

Therefore, it was hypothesized that SPC can partly replace animal protein supplements without negatively affecting intestinal immune status, intestinal oxidative stress status, nutrient digestibility, mucosa-associated microbiota, and growth performance of nursery pigs. To test the hypothesis, the objective of this study was to evaluate the effects of increasing levels of SPC supplementation replacing animal protein supplements on intestinal immune status, intestinal oxidative stress status, nutrient digestibility, mucosa-associated microbiota, and growth performance of nursery pigs.

## Materials and Methods

The procedure of this study was reviewed and approved by North Carolina State University Animal Care and Use Committee (Raleigh, NC). This experiment was conducted at the North Carolina State University Metabolism Educational Unit (Raleigh, NC).

### Antinutritional allergenic proteins in soy protein supplements

The concentration of glycinin and β-conglycinin in the soy proteins were measured using Glycinin ELISA Kit (BA-UBT002, Unibiotest, Wuhan, China) and β-conglycinin ELISA Kit (BA-UBT001, Unibiotest) following the instructions of the manufacturer. The SBM was obtained from the North Carolina State University Feed Mill Education Unit (Raleigh, NC, USA) and the SPC (X-Soy 200) was obtained from CJ Selecta (Araguari, MG, Brazil). Prior to the measurement, the soy proteins were extracted using sample extractant from the ELISA kits, then shacked vigorously for 16 h at 25 °C, centrifuged at 4,000 ×*g*, and diluted 70 folds with sample diluent. The absorbance was measured at 450 and 630 nm, and the concentration was calculated using a standard curve generated from the standard concentration and absorbance (Table 1).

**Table 1.** Composition of amino acids and antinutritional factors in soy products and animal protein supplements^1^

Item	SBM^2^	SPC^3^	Poultry meal	Fish meal	Blood plasma
Arg, %	3.45	4.59	4.05	3.84	4.39
His, %	1.28	1.67	1.32	1.44	2.53
Ile, %	2.14	2.79	2.35	2.56	2.69
Leu, %	3.62	4.68	4.25	4.47	7.39
Lys, %	2.96	3.87	3.96	4.56	6.90
Met+Cys, %	1.36	1.77	1.85	2.34	3.39
Phe, %	2.40	3.18	2.41	2.47	4.25
Thr, %	1.86	2.47	2.37	2.58	4.47
Trp, %	0.66	0.81	0.60	0.63	1.41
Val, %	2.23	3.01	2.92	3.06	5.12
Glycinin, mg/g	112.6	<0.1	–	–	–
β-conglycinin, mg/g	125.0	0.1	–	–	–

The amino acid composition of SBM, fish meal, and blood plasma were from NRC (2012); the amino acid composition of poultry meal was from Rojas and Stein (2013); the amino acid composition of SPC was from analyzed values (CJ Selecta, MG, Brazil).

SBM, soybean meal.

SPC, soy protein concentrate (X-Soy 200, CJ Selecta, MG, Brazil).

### Experimental design, animals, and diets

Thirty-two newly weaned pigs at 21 d of age with an initial BW of 6.4 ± 0.4 kg were purchased from a commercial farm (Kilpatrick Hog Farm, Magnolia, NC). Pigs were allotted four treatments in a randomized complete block design with sex (barrow and gilt) and initial BW (light and heavy) as blocking criteria. Dietary treatments were supplemented with SPC at four levels replacing animal protein supplements including fish meal, poultry meal, and blood plasma from 0% to 100%. The treatments were SPC 0% (diets with fish meal 4/2/1%, poultry meal 10/8/4%, and blood plasma 4/2/1% for phase 1/2/3, respectively); SPC 33% (SPC 0% diets with SPC replacing 33% of animal protein supplements); SPC 66% (SPC 0% diets with SPC replacing 66% of animal protein supplements); and SPC 100% (SPC 0% diets with SPC replacing 100% of animal protein supplements). The SPC 33% and SPC 66% diets were obtained by mixing different proportions of SPC 0% and SPC 100% diets (Table 2). All experimental diets were formulated to meet or exceed the nutrient requirements suggested by NRC (2012).

**Table 2.** Composition of experimental diets

Item	SPC replacement, %^1^											
	Phase 1				Phase 2				Phase 3			
	0	33	66	100	0	33	66	100	0	33	66	100
Ingredient, %												
Corn, yellow	28.99	28.22	27.45	26.68	40.67	40.48	40.29	40.10	62.57	62.48	62.40	62.31
Whey permeate	24.00	24.00	24.00	24.00	15.00	15.00	15.00	15.00	5.00	5.00	5.00	5.00
Soybean meal, 48% CP	16.00	16.00	16.00	16.00	19.00	19.00	19.00	19.00	23.00	23.00	23.00	23.00
Cookie meal	10.00	10.00	10.00	10.00	10.00	10.00	10.00	10.00	–	–	–	–
Poultry meal	10.00	6.67	3.33	–	8.00	5.33	2.67	–	4.00	2.67	1.33	–
Fish meal	4.00	2.67	1.33	–	2.00	1.33	0.67	–	1.00	0.67	0.33	–
Blood plasma	4.00	2.67	1.33	–	2.00	1.33	0.67	–	1.00	0.67	0.33	–
Soy protein concentrate	–	6.17	12.33	18.50	–	3.68	7.37	11.05	–	1.85	3.70	5.55
L-Lys HCl	0.52	0.53	0.54	0.55	0.51	0.52	0.52	0.53	0.42	0.42	0.42	0.42
L-Met	0.25	0.25	0.24	0.24	0.21	0.21	0.20	0.20	0.14	0.14	0.14	0.14
L-Thr	0.17	0.12	0.08	0.03	0.15	0.13	0.10	0.08	0.12	0.11	0.09	0.08
L-Trp	0.02	0.01	0.01	–	0.01	0.01	–	–	–	–	–	–
L-Val	–	–	–	–	–	–	0.01	0.01	–	–	–	–
Limestone	0.40	0.53	0.67	0.80	0.60	0.68	0.75	0.83	0.80	0.83	0.87	0.90
Dicalcium phosphate	–	0.28	0.57	0.85	0.20	0.45	0.70	0.95	0.55	0.67	0.78	0.90
Zinc oxide	0.25	0.25	0.25	0.25	0.25	0.25	0.25	0.25	–	–	–	–
Salt	0.22	0.22	0.22	0.22	0.22	0.22	0.22	0.22	0.22	0.22	0.22	0.22
Vitamin premix^2^	0.03	0.03	0.03	0.03	0.03	0.03	0.03	0.03	0.03	0.03	0.03	0.03
Mineral premix^3^	0.15	0.15	0.15	0.15	0.15	0.15	0.15	0.15	0.15	0.15	0.15	0.15
Poultry fat	1.00	1.23	1.47	1.70	1.00	1.20	1.40	1.60	1.00	1.10	1.20	1.30
Calculated composition, as-is												
Dry matter, %	91.2	91.4	91.5	91.6	90.7	90.7	90.8	90.8	89.7	89.7	89.7	89.8
ME, kcal/kg	3,436	3,434	3,433	3,431	3,419	3,417	3,416	3,414	3,373	3,373	3,372	3,372
Crude protein, %	24.6	24.3	23.9	23.6	22.6	22.2	21.7	21.3	20.9	20.7	20.4	20.2
SID^4^ Lys, %	1.50	1.50	1.50	1.50	1.35	1.35	1.35	1.35	1.23	1.23	1.23	1.23
SID Met+Cys, %	0.82	0.82	0.82	0.82	0.74	0.74	0.74	0.74	0.68	0.68	0.68	0.68
SID Trp, %	0.25	0.25	0.26	0.26	0.22	0.22	0.23	0.23	0.20	0.20	0.21	0.21
SID Thr, %	0.88	0.88	0.88	0.88	0.79	0.79	0.79	0.79	0.73	0.73	0.73	0.73
Ca, %	0.88	0.87	0.86	0.85	0.80	0.80	0.80	0.80	0.71	0.71	0.70	0.70
Total P, %	0.73	0.71	0.69	0.67	0.64	0.64	0.63	0.63	0.58	0.58	0.57	0.57
STTD^5^ P, %	0.50	0.48	0.47	0.45	0.41	0.41	0.40	0.40	0.33	0.33	0.33	0.33
Analyzed composition, as-is												
Dry matter, %	91.1	91.3	91. 6	91.7	90.3	90.4	90.4	90.5	88.9	89.0	89.0	88.8
Crude protein, %	24.7	24.0	23.6	22.5	22.1	21.6	20.9	20.7	20.7	20.1	20.6	19.7
Crude ash, %	6.76	6.67	6.78	6.75	6.25	6.24	6.17	6.37	4.74	4.79	4.87	4.88
Neutral detergent fiber, %	6.80	6.77	6.69	6.08	7.36	7.33	7.16	6.64	6.42	6.69	6.96	7.09
Acid detergent fiber, %	3.09	3.19	3.20	3.48	3.58	3.78	3.69	3.87	2.94	2.86	3.28	3.22
Ca, %	0.91	0.82	0.81	0.78	0.80	0.81	0.79	0.83	0.76	0.78	0.75	0.73
Total P, %	0.77	0.71	0.70	0.65	0.67	0.68	0.66	0.66	0.55	0.55	0.55	0.55

Dietary treatments were supplemented with soy protein concentrate (X-Soy 200, CJ Selecta, MG, Brazil) at four levels replacing animal protein supplements including fish meal, poultry meal, and blood plasma.

The vitamin premix provided per kilogram of complete diet: 6,614 IU of vitamin A as vitamin A acetate, 992 IU of vitamin D3, 19.8 IU of vitamin E, 2.64 mg of vitamin K as menadione sodium bisulfate, 0.03 mg of vitamin B12, 4.63 mg of riboflavin, 18.52 mg of D-pantothenic acid as calcium pantothenate, 24.96 mg of niacin, and 0.07 mg of biotin.

The trace mineral premix provided per kilogram of complete diet: 33 mg of Mn as manganous oxide, 110 mg of Fe as ferrous sulfate, 110 mg of Zn as zinc sulfate, 16.5 mg of Cu as copper sulfate, 0.30 mg of I as ethylenediamine dihydroiodide, and 0.30 mg of Se as sodium selenite.

SID, standardized ileal digestibility.

STTD P, standardized total tract digestible phosphorus.

Feed samples were collected from nine different points in each mixing batch and were sent for the analysis of nutrients at the North Carolina Department of Agriculture and Consumer Services (Raleigh, NC). Pigs were fed experimental diets for 35 days based on three phases: phase 1 for 10 d (to 7 kg BW), phase 2 for 12 d (to 11 kg BW), and phase 3 for 13 d (to 20 kg BW). Pigs were housed individually in pens (1.50 × 0.74 m) and water and feed were supplied *ad libitum*. During the last 5 d of the experiment, titanium dioxide (0.4%) was added to experimental diets as an indigestible external marker. The BW and feed intake were recorded in each phase to evaluate growth performance by measuring the average BW, ADG, ADFI, and G:F. Fecal scores were recorded everyday using a 1 to 5 scale: (1) very hard and dry stool, (2) firm stool, (3) normal stool, (4) loose stool, and (5) watery stool with no shape following Weaver and Kim (2014) and Guo et al. (2015).

### Economic analysis

The feed cost and price of ingredients were recorded in Raleigh, NC, during July 2022. Feed cost per pig was calculated as (phase 1 feed cost + phase 2 feed cost + phase 3 feed cost)/ pigs. Feed cost per weight gain was calculated as (feed cost/ pig)/(weight gain/ pig) as previously described by Soleimani et al. (2021)

### Samples collection

On day 35, all pigs were euthanized by exsanguination after the penetration of a captive bolt to the head. Mid-jejunum segments (3 m after duodenojejunal junction) were rinsed with 0.9% saline solution and collected in a 50 mL tube with 10% buffered formaldehyde. Mid-jejunal tissues were also collected and frozen in liquid nitrogen and then preserved at −80 °C for further analysis. After rinsed, the mucosal samples were collected from the mid-jejunum by scraping with microscope slides and placed in tubes (2 mL), which were subsequently frozen in liquid nitrogen and preserved at −80 °C for further analysis as described by Cheng et al (2021). Ileal digesta was obtained in a 150 mL container and placed on ice before being frozen at −20°C to determine the apparent ileal digestibility (AID) of nutrients.

### Oxidative stress and immune status

Jejunal mucosa was weighed (1 g) and suspended in 1 mL of phosphate-buffered saline (PBS, 0.01M phosphate, 0.0027M KCl, and 0.137M NaCl), then homogenized for 30 s on the ice with a tissue homogenizer (Tissuemiser; Thermo Fisher Scientific Inc, Waltham, MA). The homogenized samples were placed in new 2 mL microcentrifuge tubes and centrifuged for 10 min at 13,000 × *g* as described by Holanda and Kim (2021). The supernatants were pipetted into six aliquots and stored at −80 °C for further measurements.

The concentration of total protein, malondialdehyde (MDA), protein carbonyl, tumor necrosis factor alpha (TNF-α), immunoglobulin G (IgG), immunoglobulin A (IgA), and interleukin 8 (IL-8) were determined using commercial kits following the instruction of the manufacturer. The OD value was measured using an ELISA plate reader (Synergy HT, BioTek Instruments, Winooski, VT) and program (Gen5 Data Analysis Software, BioTek Instruments). The respective concentrations were calculated according to the absorbance of standard curves.

The homogenized mucosal supernatant was diluted (1:60) in PBS to get the required range (20 to 2,000 μg/mL), then the total protein concentration was measured by using Pierce BCA Protein Assay Kit (#23225, Thermo Fisher Scientific) as described by Holanda et al. (2020). The absorbance was read at 562 nm, the total protein concentration was used to normalize the concentration of other parameters in the mucosa.

Protein carbonyl was measured by using OxiSelect Protein Carbonyl ELISA Kit (#STA-310, Cell Biolabs, San Diego, CA). All samples were diluted in PBS to reach the protein concentration at 10 μg/mL before measurement. The range of standard was 0.375 to 7.500 nmol/mg protein. All processes were carried out according to the instructions of the manufacturer. The absorbance was measured at 450 nm and the concentration was represented as nmol/mg protein.

The concentration of MDA in the mucosa was determined using OxiSelect TBARS MDA Quantitation Assay Kit (#STA-330, Cell Biolabs). The working range of the standard is from 0 to 125 μM. The absorbance was measured at 532 nm. The concentration was calculated based on the absorbance value of the standard and represented as nmol/mg protein.

The concentration of IgA and IgG was measured using the ELISA kits (E101-102 and E101-104, Bethyl Laboratories, Montgomery, TX) as described by Holanda et al. (2020) and Duarte et al. (2020). The mucosal supernatants were diluted in PBS to 1:1,200 and 1:2,400, respectively to get the required working range for measurement. Absorbance was read at 450 nm and the concentration was represented as μg/mg of protein.

The concentration of TNF-α was measured using the Porcine TNF-α Immunoassay Kit (#PTA00, R&D Systems, Minneapolis, MN) as described by Sun et al. (2021). Absorbance was read at 450 nm and corrected at 570 nm. The concentration of TNF-α was represented as pg/mg protein.

The concentration of IL-8 was measured by using Porcine IL-8/CXCL8 Quantikine ELISA kit (#P8000, R&D Systems) as described by Moita et al. (2021). All samples were diluted in reagent diluent to 1:5 to measure. The absorbance was read at 450 nm and corrected at 570 nm. The concentration was represented as pg/mg protein.

### Intestinal anorectic hormone

Mid-jejunal tissue was weighed (0.2 g) and suspended in 2 mL of Tissue Extraction Reagent (FNN0071, Thermo Fisher Scientific) with Protease Inhibitor (P2714, Sigma-Aldrich, St. Louis, MO), then homogenized on the ice with a tissue homogenizer (Tissuemiser; Thermo Fisher Scientific Inc.). The homogenized samples were placed in new 2 mL microcentrifuge tubes and centrifuged for 5 min at 10,000 ×*g*. The supernatants were collected and stored at −80 °C for further measurements.

The concentration of PYY was measured by using Pig Peptide YY ELISA Kit (RK07593, ABclonal Technology, Woburn, MA) following the instruction of the manufacturer. The absorbance was read at 450 nm and corrected at 570 nm. The concentration was represented as pg/mL protein.

### Intestinal morphology and enterocyte proliferation

Two sections of the mid-jejunum were fixed in 10% formalin for two days and then moved to a 70% ethanol solution. Embedment, staining, and dehydration were performed at the North Carolina State University Histology Laboratory (College of Veterinary Medicine, Raleigh, NC). Automated Ki-67 stain was performed on Biocare Intellipath stainer (Biocare Medical, Pacheco, CA). A primary monoclonal antibody of Ki-67 (#ACR325, Biocare Medical) was used after 1:100 dilution with 30 min incubation at room temperature. Vector ImmPress Rabbit polymer was used for detection. Diaminobenzamine (DAB) as a chromogen was used for staining. Villus height, villus width, and crypt depth were measured using a microscope Olympus CX31 at 40×(Lumenera Corporation, Ottawa, Canada) and Infinity 2-2 digital CCD software. Ten intact villi and their related crypts were measured in each slide. The villus height was measured from the top of the villus to the junction of villus and crypt; the villus width was measured at the middle portion of the villus; and the crypt depth was measured from the junction of villus and crypt to the bottom of the crypt. The ratio of villus height to crypt depth (VH: CD) was determined by dividing villus height by crypt depth. The proportion of Ki-67 positive cells as a predictor of proliferating enterocytes was measured using images of 10 intact crypts taken by microscope Olympus CX31 at 100×. The cropped images were analyzed with Image JS and processed by the same person.

### Apparent ileal digestibility

Ileal digesta was freeze-dried by freeze drier for 48 h (24D 48, Virtis, Gardiner, NY). Dried feed and ileal digesta were used for further analysis. The dry matter (DM), method (930.15), and ether extract (EE), method (2003.06) were measured based on AOAC (2007). Gross energy (GE) was measured using a bomb calorimeter (Model 6200, Parr Instrument Company, Moline, IL). The concentration of crude protein (CP) and amino acids (AA) in feed and digesta samples were measured at Experiment Station Chemical Laboratories of the University of Missouri-Columbia. The concentration of titanium dioxide in the feed and digesta was calculated following Myers et al. (2004). The apparent ileal digestibility (AID) of DM, GE, EE, CP, and AAs was calculated by using the following function as described by Chen et al. (2020):


$$\begin{array}{ll} AID\;\left(\%\right)\;= & \;\{1\;[\left(TiO_{2feed}/\;TiO_{2digesta}\right)\;\\ & *\;\left(Nutrient_{digesta}/\;Nutrient_{feed}\right)]\}\;*\;100 \end{array}$$

In which TiO_2feed_ and TiO_2digesta_ were the measured concentration of titanium dioxide in the feed and in the digesta, respectively; Nutrient_digesta_ and Nutrient_feed_ were the measured concentration of nutrient in the digesta and in the feed, respectively.

### Relative abundance and diversity of jejunal mucosa-associated microbiota

The DNA in jejunal mucosa was extracted using QIAamp Fast DNA Stool kit (#51604, Qiagen, Germantown, MD). The extracted DNA was sent to Mako Medical Laboratories (Raleigh, NC) to analyze microbiota sequencing using the 16S rRNA technique. First, samples were prepared using Ion Chef equipment for the template, then analyzed on the Ion S5 system (Thermo Fisher Scientific). Different sequences V2, V3, V4, V6, V7, V8, and V9 were amplified using Ion 16S Metagenomics Kit 113 (Thermo Fisher Scientific) and these sequences were analyzed using Torrent Suite Software (version 5.2.2) to get raw unaligned sequence data files. Then microbial analysis including alignment to GreenGenes and MicroSeq databases, and OTU table generation were conducted using Ion Reporter Software Suite (version 5.2.2) of bioinformatics analysis tools (Thermo Fisher Scientific). Finally, sample analyses were performed by using Ion Reporter’s Metagenomics 16S workflow powered by Qiime (version w1.1). The microbial diversity was evaluated by alpha-diversity (Chao1, Shannon, and Simpson) and beta-diversity (Bray–Curtis) distance.

### Statistical analysis

Data were analyzed with the MIXED procedure in SAS 9.4 (SAS Inc., Cary, NC). The main effect was dietary treatment, considered as a fixed effect, and initial BW and sex blocks were considered as random effects. The number of replications was determined based on a power test (Martin et al., 1987) to determine the effects of increasing SPC supplementation by replacing animal protein supplements. The experimental unit was the pig that was housed and fed individually. The linear and quadratic effects of increasing SPC supplementation by replacing animal protein supplements were tested by polynomial contrasts. The means were calculated using the LSMEANS statement in SAS. A contrast was performed using the CONTRAST statement to evaluate the effects of SPC supplementation (SPC 0% vs. others). The exponential regression was fitted using the NLIN procedure of SAS to estimate the level of animal protein supplements that can be replaced by SPC without affecting the growth performance of nursery pigs. The following nonlinear equation was applied:


$$\begin{array}{l} y\;=\;a\;+\;b\;*\;\left(1\;+\;e^{\left(c\;*\;x\right)}\right), \end{array}$$

in which y = performance criterion (average daily gain, feed efficiency); a = intercept (growth performance); b = asymptotic response; a + b = common asymptote (maximum growth performance); c = steepness coefficient for level of animal protein supplements replaced by SPC; x = level of animal protein supplements replaced by SPC.

For the microbiota data, a contrast was performed to evaluate the effect of SPC supplementation on the relative abundance and alpha diversity of mucosa-associated microbiota (SPC 0% vs. SPC 100%). The analysis of similarities (ANOSIM) was performed to evaluate the beta diversity of mucosa-associated microbiota. The data were “visualized” using principal coordinates analysis (PCoA) based on Bray-Curtis distance. The *P* value less than 0.05 was considered as the statistical significance and *P* value between 0.05 and 0.10 was considered as tendency.

## Results

### Growth performance and fecal score

**Figure 1.**
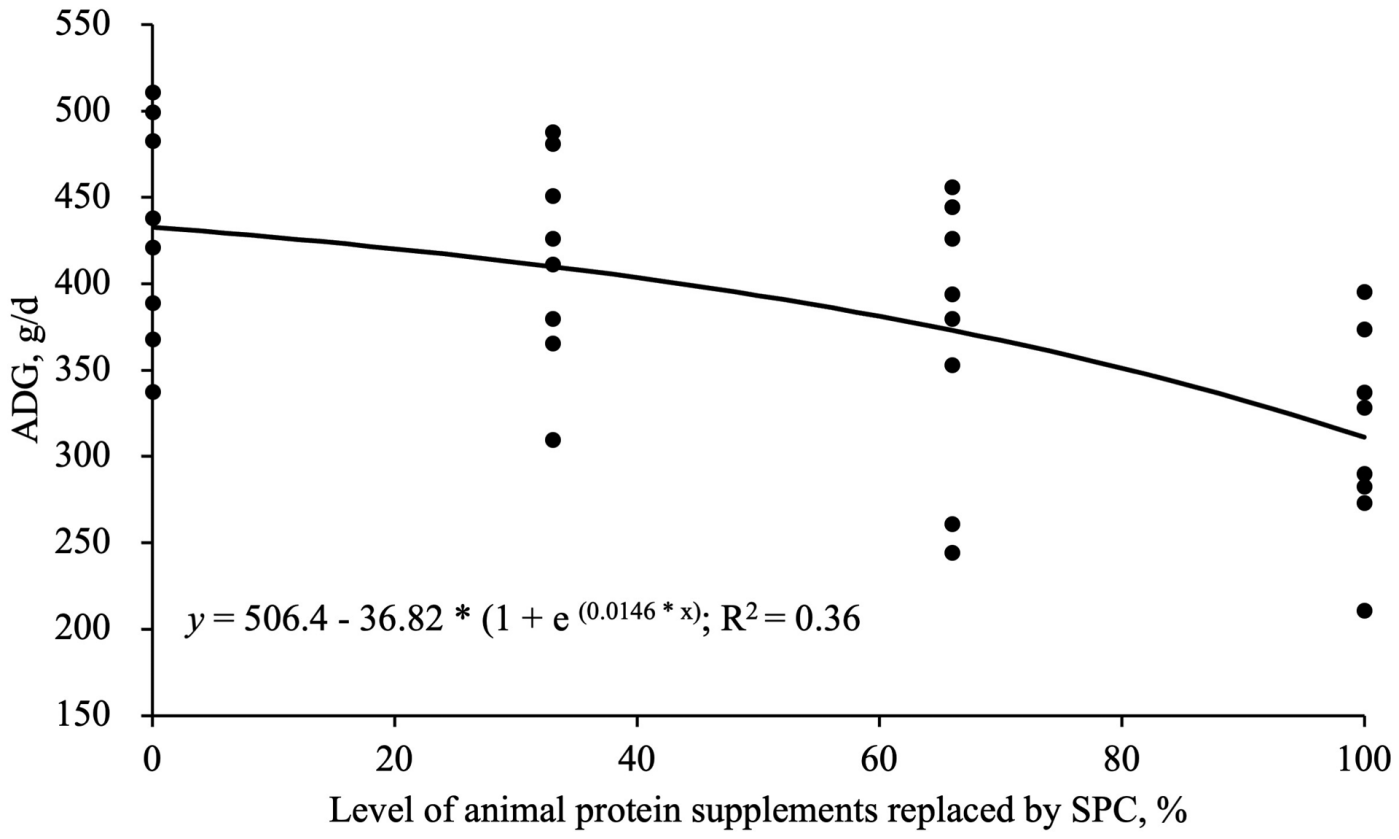
The ADG of nursery pigs fed diets with the level of SPC replacing animal protein supplements in overall. Based on an exponential model, the 95% maximum response was obtained at 10.5% animal protein supplements replaced by SPC; The equation is: ADG (g/d) = 506.4 − 36.82 * (1 + e ^(0.0146 * x)^) (*P* < 0.05).

**Figure 2.**
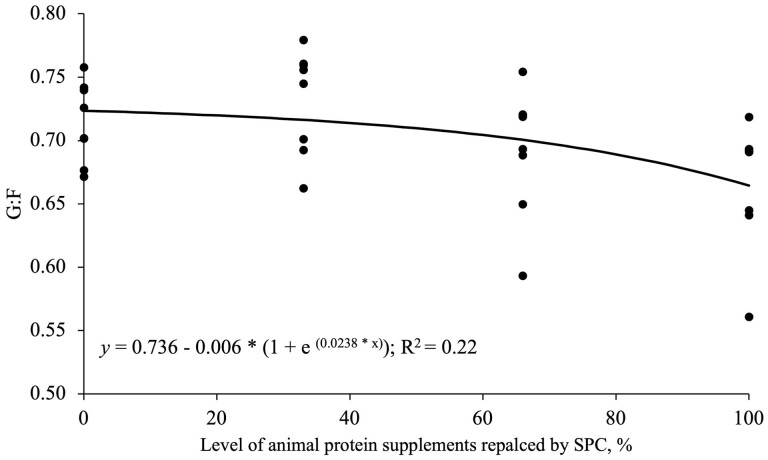
The G:F of nursery pigs fed diets with the level of SPC replacing animal protein supplements in overall. Based on an exponential model, the 95% maximum response was obtained at 16.8% animal protein supplements replaced by SPC; The equation is: y (G:F) = 0.736 − 0.006 * (1 + e ^(0.0238 * x)^) (*P* < 0.05).

Increasing SPC supplementation by replacing animal protein supplements linearly reduced (*P* < 0.05) BW, ADG, and ADFI during all experimental phases and overall (Table 3). Increasing SPC supplementation by replacing animal protein supplements decreased linearly (*P* < 0.05) G:F in phase 1 and overall (Table 3). An exponential regression analysis showed that SPC supplementation can replace (*P* < 0.05) animal protein supplements up to 10.5%, and 16.5% without reducing the ADG, and G:F of the nursery pigs, respectively (Figures 1 and 2). The fecal score was not affected by increasing SPC supplementation by replacing animal protein supplements (Table 3).

**Table 3.** Growth performance and fecal score of nursery pigs fed diets with supplementation of soy protein concentrate (SPC) replacing animal protein supplements

Item	SPC replacement, %^1^				SEM	*P* value		
	0	33	66	100		Linear	Quad.	SPC 0% vs. others
BW, kg								
day 0	6.3	6.4	6.4	6.4	0.4	0.807	0.964	0.854
day 10	7.9	7.8	7.4	6.9	0.4	0.015	0.568	0.101
day 22	12.5	12.6	11.6	10.3	0.9	0.003	0.218	0.098
day 35	21.4	20.9	19.3	17.3	1.2	0.001	0.376	0.021
ADG, g/d								
Phase 1^2^	159	139	106	51	27	0.007	0.522	0.065
Phase 2^3^	384	406	345	286	44	0.009	0.160	0.255
Phase 3^4^	684	633	596	535	38	0.006	0.888	0.029
Overall	431	414	370	312	27	0.001	0.379	0.020
ADFI, g/d								
Phase 1	211	197	180	126	24	0.016	0.396	0.128
Phase 2	498	498	458	386	50	0.023	0.317	0.216
Phase 3	1,001	912	874	798	55	0.003	0.887	0.012
Overall	603	566	533	465	39	0.002	0.585	0.020
G:F								
Phase 1	0.72	0.68	0.67	0.52	0.09	0.042	0.357	0.192
Phase 2	0.78	0.81	0.77	0.73	0.04	0.318	0.378	0.904
Phase 3	0.68	0.70	0.68	0.67	0.02	0.534	0.615	0.927
Overall	0.72	0.73	0.69	0.66	0.02	0.013	0.188	0.332
Fecal score								
Phase 1	3.3	3.5	3.7	3.6	0.16	0.226	0.349	0.161
Phase 2	3.2	3.1	3.3	3.3	0.07	0.155	0.707	0.552
Phase 3	3.0	3.0	3.0	3.1	0.02	0.399	0.122	0.639

Dietary treatments were supplemented with soy protein concentrate (X-Soy 200, CJ Selecta, MG, Brazil) at four levels replacing animal protein supplements including fish meal, poultry meal, and blood plasma (*N* = 32 total, *n* = 8 for each replacement level).

Phase 1, from day 0 to day 10.

Phase 2, from day 10 to day 22.

Phase 3, from day 22 to day 35.

### Economic analysis

Increasing SPC supplementation by replacing animal protein supplements linearly reduced (*P* < 0.05) feed cost/pigs and feed cost/kg gain (Table 4).

**Table 4.** Economic analysis of nursery pigs fed diets with supplementation of soy protein concentrate (SPC) replacing animal protein supplements

Item	SPC replacement, %^1^				SEM	*P* value		
	0	33	66	100		Linear	Quad.	SPC 0% vs. others
Feed cost/ pig, $^2^	11.99	10.73	9.50	7.71	0.74	<0.001	0.645	<0.001
Feed cost/ kg gain, $^3^	0.80	0.74	0.74	0.72	0.02	0.009	0.402	0.008

Dietary treatments were supplemented with soy protein concentrate (X-Soy 200, CJ Selecta, MG, Brazil) at four levels replacing animal protein supplements including fish meal, poultry meal, and blood plasma (*N* = 32 total, *n* = 8 for each replacement level).

Feed cost/pig = (phase 1 cost + phase 2 cost + phase 3 cost)/pig.

Feed cost/kg gain = (feed cost/pig)/(weight gain/ pig).

### Oxidative stress, immune status, intestinal anorectic hormone, intestinal morphology, and enterocyte pproliferation

Increasing SPC supplementation by replacing animal protein supplements did not affect the oxidative stress in jejunal mucosa. Supplementation of SPC replacing animal protein supplements tended to increase (*P* = 0.099) the concentration of IgA in the jejunal mucosa of nursery pigs compared to no supplementation of SPC (Table 5). Increasing SPC supplementation by replacing animal protein supplements did not affect the concentration of MDA, TNF-α, IL-8, protein carbonyl, and IgG in the jejunum (Table 5).

**Table 5.** Oxidative stress, immune status, peptide tyrosine tyrosine (PYY), intestinal morphology, and enterocyte proliferation of nursery pigs fed diets with supplementation of soy protein concentrate (SPC) replacing animal protein supplements

Item	SPC replacement, %^1^				SEM	*P* value		
	0	33	66	100		Linear	Quad.	SPC 0% vs. others
Jejunal mucosa,/mg of protein								
Protein carbonyl, nmol	1.38	1.46	0.81	1.17	0.32	0.314	0.612	0.462
Malondialdehyde, nmol	0.83	0.89	0.87	0.82	0.13	0.962	0.694	0.829
IgA^2^, μg	3.39	6.44	4.68	5.54	1.06	0.337	0.309	0.099
IgG^3^, μg	2.91	2.88	2.58	2.52	0.50	0.525	0.970	0.659
IL-8^4^, pg	343	329	314	339	45	0.896	0.674	0.768
TNF-α^5^, pg	0.35	0.27	0.36	0.30	0.07	0.800	0.873	0.548
Jejuanl tissue, mg of protein								
PYY, pg	1.13	1.19	1.34	1.49	0.18	0.015	0.666	0.096
Intestinal morphology								
Villus height, μm	529	535	546	518	45	0.881	0.596	0.912
Villus width, μm	109	108	104	117	9	0.559	0.440	0.894
Crypt depth, μm	297	282	294	269	13	0.241	0.694	0.343
VH:CD6	1.81	1.94	1.89	1.94	0.18	0.598	0.774	0.497
Enterocyte proliferation								
Ki-67 positive^7^, %	32.2	29.9	30.8	28.9	1.5	0.150	0.874	0.147

Dietary treatments were supplemented with soy protein concentrate (X-Soy 200, CJ Selecta, MG, Brazil) at 4 levels replacing animal protein supplements including fish meal, poultry meal, and blood plasma (*N* = 32 total, *n* = 8 for each replacement level).

IgA, immunoglobulin A.

IgG, immunoglobulin G.

IL-8, interleukin 8.

TNF-α, tumor necrosis factor alpha.

VH:CD, villus height to crypt depth ratio.

Ki-67 positive, enterocyte proliferation rate in the crypt.

Increasing SPC supplementation by replacing animal protein supplements linearly increased (*P* < 0.05) the concentration of PYY in jejunal tissue. Supplementation of SPC replacing animal protein supplements tended to increase (*P* = 0.096) the concentration of PYY in the jejunal tissue of nursery pigs compared no supplementation of SPC (Table 5).

Increasing SPC supplementation replacing animal protein supplements did not affect villus height, villus width, crypt depth, VH:CD ratio, and enterocyte proliferation (Table 5).

### Apparent ileal digestibility

Increasing SPC supplementation replacing animal protein supplements did not affect AID of DM, GE, EE, CP, and AA (Table 6).

**Table 6.** Apparent ileal digestibility of nursery pigs fed diets with supplementation of soy protein concentrate (SPC) replacing animal protein supplements

Item, %	SPC replacement, %^1^				SEM	*P* value		
	0	33	66	100		Linear	Quad.	SPC 0% vs. others
Dry matter	55.4	61.3	57.3	61.2	5.2	0.471	0.802	0.345
GE	50.7	62.7	52.3	57.9	4.4	0.532	0.425	0.144
Ether extract	70.2	65.4	65.9	74.9	5.9	0.564	0.242	0.805
Crude protein	62.1	69.9	61.2	69.7	3.5	0.326	0.903	0.186
Lys	74.9	80.5	69.6	79.4	2.5	0.830	0.421	0.589
Met+Cys	62.9	71.6	61.1	72.4	4.0	0.256	0.670	0.173
Trp	69.2	76.6	65.4	74.0	3.3	0.849	0.859	0.477
Thr	59.7	68.0	55.7	65.4	4.3	0.785	0.859	0.458
Val	61.7	70.4	57.5	68.4	3.8	0.645	0.745	0.352
Ile	66.3	74.2	62.9	73.0	3.3	0.511	0.690	0.275
Leu	64.3	72.6	62.7	71.2	4.3	0.465	0.981	0.227
Phe	66.8	75.4	65.5	73.7	3.5	0.390	0.951	0.145
His	67.9	76.1	65.6	74.0	3.3	0.511	0.986	0.189
Arg	77.7	83.6	75.5	82.2	2.2	0.532	0.850	0.217

Dietary treatments were supplemented with soy protein concentrate (X-Soy 200, CJ Selecta, MG, Brazil) at four levels replacing animal protein supplements including fish meal, poultry meal, and blood plasma (*N* = 32 total, *n* = 8 for each replacement level).

### Relative abundance and diversity of jejunal mucosa-associated microbiota

**Figure 3.**
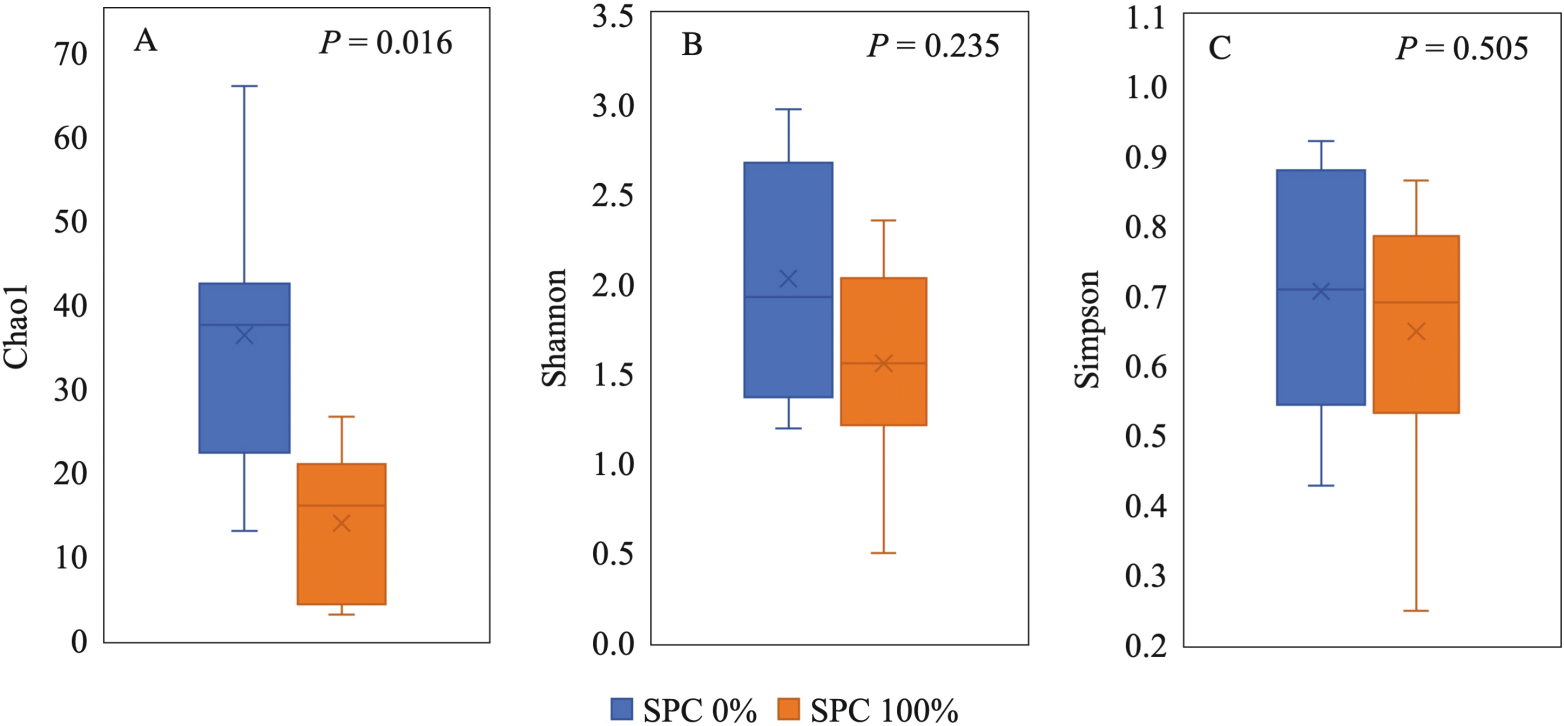
The alpha diversity of mucosa-associated microbiota estimated with Chao1 (A), Shannon (B), and Simpson (C) indexes. (Chao1: SPC 0% vs. SPC 100%, *P* < 0.05).

**Figure 4.**
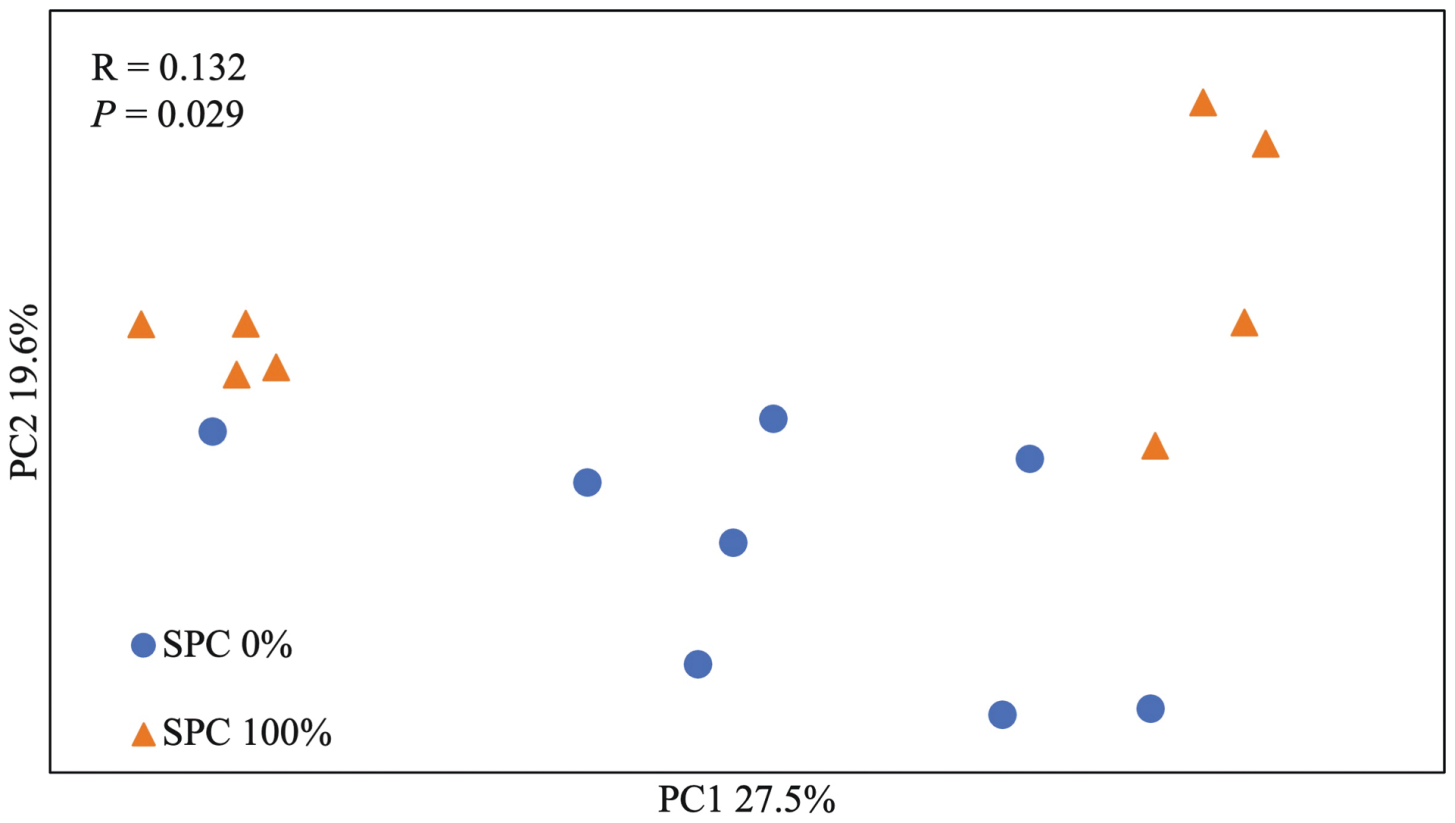
Beta diversity comparison for each treatment. Principal coordinates analysis (PCoA) based on Bray–Curtis distance was presented. The analysis of similarity (ANOSIM) procedure was used for significance of clustering pattern between SPC 0% and SPC 100% treatment.

At the phylum level (Table 7), supplementation of SPC 100% did not affect the relative abundance of microbiota. At the family level (Table 8), supplementation of SPC 100% decreased (*P* < 0.05) the relative abundance of *Helicobacteraceae*, *Campylobacteraceae*, *Corynebacteriaceae*, *Staphylococcaceae*, *Bradyrhizobiaceae*, and *Bacillaceae* compared with no supplementation of SPC. At genus level (Table 9), supplementation of SPC 100% tended to increase (*P* = 0.085) the relative abundance of *Pelomonas*, whereas it decreased (*P* < 0.05) the relative abundance of *Corynebacterium*, *Staphylococcus*, and *Bacillus*, and tended to decrease (*P* = 0.065) the relative abundance of *Campylobacter*. At the species level (Table 10), supplementation of SPC 100% increased (*P* < 0.05) the relative abundance of *Pelomonas aquatic* and tended to increase (*P* = 0.055) relative abundance of *Pelomonas puraquae*, whereas it decreased (*P* < 0.05) the relative abundance of *Helicobacter rappini*. The alpha diversity of mucosa-associated microbiota showed that supplementation of SPC replacing all animal protein supplements decreased (*P* < 0.05) Chao1 index compared with no supplements (*P* < 0.05). However, there was no difference between Shannon and Simpson (Figure 3). The microbial community was visualized using PCoA based on Bray-Curtis distance, which confirmed that the supplementation of SPC replacing animal protein supplements in the diets changed (*R* = 0.13, *P* < 0.05) microbiota composition in jejunal mucosa of nursery pigs (Figure 4).

**Table 10.** Relative abundance of jejunal mucosa-associated microbiota at species level in nursery pigs fed diets with supplementation of soy protein concentrate (SPC) completely replacing animal protein supplements

Item	SPC replacement, %^1^		SEM	*P* value
	0	100		SPC 0% vs. SPC 100%
*Helicobacter rappini*	28.50	4.27	9.93	0.045
*Prevotella copri*	12.30	24.81	14.98	0.191
*Alcaligenes faecalis*	6.00	<0.01	4.24	0.337
*Chlamydia suis*	4.35	6.80	6.79	0.758
*Pelomonas puraquae*	3.03	11.08	3.44	0.055
*Facklamia ignava*	1.87	<0.01	1.05	0.130
*Propionibacterium acnes*	1.70	9.48	4.06	0.153
*Microbacterium ginsengisoli*	1.52	2.46	1.58	0.552
*Pelomonas aquatica*	1.36	6.40	2.87	0.044
*Helicobacter equorum*	1.24	1.33	1.30	0.961
*Roseburia faecis*	1.05	2.68	2.08	0.283
*Corynebacterium imitans*	0.95	<0.01	0.39	0.016
*Helicobacter mastomyrinus*	0.88	5.41	3.55	0.318
*Bifidobacterium boum*	0.80	1.84	1.36	0.383
*Prevotella stercorea*	0.76	2.21	1.39	0.264
*Succinivibrio dextrinosolvens*	0.74	0.30	0.46	0.508
*Campylobacter coli*	0.72	<0.01	0.45	0.222
*Mitsuokella jalaludinii*	0.61	0.28	0.23	0.322
*Prevotella ruminicola*	0.59	0.09	0.33	0.270
*Cupriavidus necator*	0.50	1.02	0.72	0.425
*Clostridium butyricum*	0.40	1.12	0.85	0.506
*Lactobacillus mucosae*	0.36	2.16	1.35	0.365
*Helicobacter sp.*	0.24	<0.01	0.14	0.249
*Dialister succinatiphilus*	0.20	1.52	0.92	0.184
*Lactobacillus ruminis*	0.16	0.05	0.08	0.174
Others	29.18	14.51	7.98	0.159

Dietary treatments were supplemented with soy protein concentrate (X-Soy 200, CJ Selecta, MG, Brazil) replacing animal protein supplements including fish meal, poultry meal, and blood plasma (*N* = 16 total, *n* = 8 for each replacement level).

**Table 7.** Relative abundance of jejunal mucosa-associated microbiota at phylum level in nursery pigs fed diets with supplementation of soy protein concentrate (SPC) completely replacing animal protein supplements

Item	SPC replacement, %^1^		SEM	*P* value
	0	100		SPC 0% vs. SPC 100%
Proteobacteria	64.22	42.34	17.58	0.132
Firmicutes	11.91	21.84	8.24	0.299
Bacteroidetes	11.37	19.89	13.38	0.352
Actinobacteria	8.58	10.91	4.41	0.712
Chlamydiae	2.36	4.61	4.29	0.661
Spirochaetes	0.6	0.11	0.41	0.313
Others	0.96	0.29	0.52	0.343

Dietary treatments were supplemented with soy protein concentrate (X-Soy 200, CJ Selecta, MG, Brazil) replacing animal protein supplements including fish meal, poultry meal, and blood plasma (*N* = 16 total, *n* = 8 for each replacement level).

**Table 8.** Relative abundance of jejunal mucosa-associated microbiota at family level in nursery pigs fed diets with supplementation of soy protein concentrate (SPC) completely replacing animal protein supplements

Item	SPC replacement, %^1^		SEM	*P* value
	0	100		SPC 0% vs. SPC 100%
*Helicobacteraceae*	25.77	3.02	8.87	0.042
*Prevotellaceae*	10.29	18.94	13.07	0.320
*Moraxellaceae*	8.61	0.01	4.04	0.158
*Alcaligenaceae*	5.82	<0.01	4.04	0.328
*Comamonadaceae*	4.28	13.04	6.41	0.152
*Campylobacteraceae*	3.62	0.13	1.08	0.040
*Veillonellaceae*	3.03	3.50	2.26	0.746
*Corynebacteriaceae*	2.53	<0.01	0.92	0.040
*Pseudomonadaceae*	2.21	6.64	4.96	0.499
*Xanthomonadaceae*	1.69	0.15	1.06	0.290
*Clostridiaceae*	1.53	4.61	1.91	0.277
*Enterobacteriaceae*	1.34	13.41	7.58	0.256
*Sphingomonadaceae*	1.33	0.17	0.54	0.157
*Microbacteriaceae*	1.32	3.35	2.15	0.461
*Staphylococcaceae*	1.31	0.06	0.26	0.003
*Propionibacteriaceae*	1.22	5.20	2.50	0.251
*Aerococcaceae*	1.22	<0.01	0.65	0.105
*Lactobacillaceae*	1.09	8.15	3.69	0.169
*Burkholderiaceae*	1.05	0.78	0.57	0.622
*Lachnospiraceae*	1.02	1.80	1.30	0.321
*Bifidobacteriaceae*	1.00	2.05	1.46	0.448
*Succinivibrionaceae*	0.93	2.76	1.50	0.376
*Methylobacteriaceae*	0.69	0.58	0.38	0.835
*Ruminococcaceae*	0.57	0.54	0.38	0.927
*Brachyspiraceae*	0.55	<0.01	0.36	0.265
*Rhodobacteraceae*	0.51	0.03	0.25	0.191
*Caulobacteraceae*	0.48	0.71	0.68	0.745
*Micrococcaceae*	0.48	0.01	0.21	0.110
*Bradyrhizobiaceae*	0.31	<0.01	0.07	0.007
*Streptococcaceae*	0.22	1.23	0.65	0.231
*Bacillaceae*	0.15	0.01	0.05	0.003
Others	13.82	9.11	6.34	0.438

Dietary treatments were supplemented with soy protein concentrate (X-Soy 200, CJ Selecta, MG, Brazil) replacing animal protein supplements including fish meal, poultry meal, and blood plasma (*N* = 16 total, *n* = 8 for each replacement level).

**Table 9.** Relative abundance of jejunal mucosa-associated microbiota at genus level in nursery pigs fed diets with supplementation of soy protein concentrate (SPC) completely replacing animal protein supplements

Item	SPC replacement, %^1^		SEM	*P* value
	0	100		SPC 0% vs. SPC 100%
*Helicobacter*	29.00	8.01	8.76	0.116
*Prevotella*	10.92	19.76	14.24	0.361
*Acinetobacter*	7.19	<0.01	4.32	0.262
*Alcaligenes*	4.78	<0.01	3.38	0.337
*Pelomonas*	4.62	17.01	5.61	0.085
*Campylobacter*	3.54	0.19	1.17	0.065
*Chlamydia*	3.04	5.25	5.07	0.712
*Corynebacterium*	3.02	<0.01	1.09	0.043
*Pseudomonas*	2.00	8.06	5.21	0.378
*Clostridium*	1.68	5.15	2.29	0.306
*Microbacterium*	1.51	3.53	2.20	0.479
*Staphylococcus*	1.40	0.08	0.32	0.005
*Facklamia*	1.32	0.01	0.73	0.120
*Propionibacterium*	1.26	5.73	2.68	0.210
*Bifidobacterium*	1.11	2.52	1.74	0.383
*Lactobacillus*	1.00	8.93	3.76	0.161
*Succinivibrio*	0.94	2.78	1.63	0.392
*Selenomonas*	0.92	0.08	0.56	0.229
*Mitsuokella*	0.88	0.56	0.36	0.518
*Methylobacterium*	0.81	0.63	0.40	0.753
*Cupriavidus*	0.63	0.58	0.40	0.922
*Sphingomonas*	0.59	<0.01	0.18	0.039
*Ralstonia*	0.48	0.28	0.22	0.535
*Brevundimonas*	0.44	0.12	0.29	0.151
*Arthrobacter*	0.44	<0.01	0.20	0.119
*Streptococcus*	0.26	1.48	0.75	0.205
*Bacillus*	0.14	<0.01	0.06	0.028
Others	16.07	9.27	5.23	0.197

Dietary treatments were supplemented with soy protein concentrate (X-Soy 200, CJ Selecta, MG, Brazil) replacing animal protein supplements including fish meal, poultry meal, and blood plasma (*N* = 16 total, *n* = 8 for each replacement level).

## Discussion

Antinutritional factors in the soybean restrict its use in feeds due to their adverse effects on the intestinal immune response of nursery pigs (Li et al., 1991a). The heat processing in SBM production could efficiently inactivate several antinutritional factors, such as trypsin inhibitors and lectins (Fasina et al., 2003; Peres et al., 2003). However, two soy antigens in SBM, glycinin, and β-conglycinin, are resistant to heat processing and can be involved in the hypersensitive reaction and growth depression in nursery pigs (Li et al., 1991b; Sun et al., 2008b; Hao et al., 2009). Ethanol extraction has been used to remove soluble carbohydrates and reduce the content of soy allergenic proteins while retaining a relatively high CP content compared to SBM (Peisker, 2001). This study showed that ethanol processing reduced the antigenicity of glycinin and β-conglycinin in SBM, which was in accordance with Peisker (2001). The reduction of these two soy antigens is possibly due to the substantial structural rearrangements caused by ethanol denaturation, resulting in the loss of antibody binding epitopes (Moreira et al., 1981; Sissons et al., 1982). Therefore, ethanol extraction could be an effective way to reduce the negative impacts of SBM by reducing the contents of soy antigens.

In this study, pigs were housed individually to determine the changes in growth performance, immune status, oxidative stress status, intestinal morphology, nutrient digestibility, mucosa-associated microbiota of nursery pigs by measuring the accurate SPC intake of pigs as suggested by previous studies (Jang and Kim, 2019; Moita et al., 2022; Xu et al., 2022). However, various environmental factors including housing patterns, interaction with other feedstuffs in feeds and ages could affect the impacts of SPC on intestinal oxidative stress, intestinal immune status, intestinal morphology, and nutrient digestibility of nursery pigs. Previous studies showed that group housing could affect physiological response, behaviors, and intestinal immune response and intestinal microbiota of pigs (Bruininx et al., 2002; Wen et al., 2021). Jang et al. (2021) also described that the status of intestinal maturation of pigs weaned at different ages may affect the dietary needs of feedstuff for the growth of nursery pigs. Interestingly, according to Lenehan et al. (2007), increasing supplemental levels of SPC replacing only SBM in nursery feeds showed a plateau at 21.4% on BW gain and feed efficiency of nursery pigs under group housing, but there was limited information about the impacts of SPC on intestinal immune status and oxidative stress, intestinal morphology, nutrient digestibility. In addition, SPC was replacing animal protein sources, not solely replacing the certain types of animal protein, as the main effect causing changes in growth and jejunal mucosa-associated microbiota of the pigs. Thus, some of the benefits could also be related to the feedstuff changes in levels of other animal protein sources including fish meal and poultry meal. However, for the complete understanding, it warrants further investigation whether SPC can effectively replace certain types of animal protein supplements without affecting intestinal immune status, intestinal oxidative stress, intestinal morphology, nutrient digestibility, and growth performance of nursery pigs.

The growth performance of pigs was reduced by the increasing supplementation of SPC in the diets during the first phase of post-weaning period. It can be speculated that the reduced feed efficiency and feed intake may have resulted from the reduction of functional compounds from animal protein supplements, which could prove more effective in phase 1. In phases 2 and 3, the impaired BW gain can be mainly attributed to the reduced feed intake due to the unchanged feed efficiency. The palatability of the diets could potentially result in the reduction of feed intake. With the reduction of high appetitive animal protein supplements, such as fish meal, blood plasma, and poultry meal, the feed intake of nursery pigs can be negatively affected. Van Dijk et al. (2001) have shown that blood plasma has a positive effect on the stimulation of feed intake of nursery pigs, especially during the first week of the post-weaning period. Previous studies have shown that pigs fed diets with fish meal and poultry meal had similar feed intake compared with the diet with blood plasma (Kim and Easter, 2001; Keegan et al., 2004). In support of the findings in this study, a previous study has shown that nursery pigs preferred to eat an SBM-based diet instead of a high amount of SPC in the diet (Lenehan et al., 2007), providing one possible explanation for the observed negative impacts on feed intake by replacing highly palatable animal protein supplements with SPC. Furthermore, feed intake is highly related to the secretion of anorectic hormones in enteroendocrine cells, such as cholecystokinin (CCK), glucagon-like peptide 1 (GLP-1), and PYY (Westerterp-Plantenga et al., 2009; Santos-Hernández et al., 2018). In addition, the effect of PYY has been shown to have potent and acute effects, which influence satiety and inhibit the feed intake of pigs (Ito et al., 2006). Soybean protein hydrolysate has been indicated to stimulate anorectic hormone secretion and inhibit feed intake in pigs via calcium-sensing receptors and intracellular calcium signaling (Wang et al., 2021). In the current study, increasing SPC supplementation increased PYY concentration in the jejunum, leading to the reduction of feed intake of pigs. Even though the SPC supplementation impaired the growth performance of nursery pigs, the economic benefit was increased because SPC is cost saving ingredient compared with animal protein supplements in nursery diets. The exponential model has also been used to estimate the optimal level in dose-response studies (Robbins et al., 1979). According to the exponential model, 10.5% or 16.8% of animal protein supplements replaced by SPC in the diets can be acceptable without negatively affecting the BW gain or feed intake of nursery pigs, respectively. This can be explained by the high protein content and amino acid profiles in SPC that meet the nutrient requirements for the growth of nursery pigs.

The benefits of using animal protein supplements in nursery diets, including fish meal, blood plasma, and poultry meal, is due to the free of antigens or antinutritional factors. In particular, blood plasma contains various functional compounds and previous studies showed that its immunoglobulin and glycoproteins contents could enhance immunity by preventing the adhesion of pathogens to the intestinal mucosa (Coffey and Cromwell, 1995; Nollet et al., 1999). Peace et al. (2011) also indicated that the inclusion of blood plasma in the nursery diet has beneficial effects on intestinal barrier function and diarrhea in weaned pigs. This reduction of blood plasma also may induce the increased IgA content in jejunal mucosa in this study. Cheng et al. (2021) reported that pigs fed reduced blood plasma from 4.08% to 3.08% resulted in increased IgA in jejunal mucosa, which supports the current result.

The changes in mucosa-associated microbiota can partly explain the increased immune response. Fouhse et al. (2016) indicated that the higher diversity of microbiota in the intestine was related to improved immunological functions, which is in agreement with the current microbiota results. The intestinal microbiota is highly related to the immune system development of pigs (Schokker et al., 2015; Jang et al., 2020). As a frontline defender, mucosa-associated microbiota plays an important role against exogenous pathogens (Isaacson and Kim, 2012). In this study, the pigs in SPC replacement significantly lowered Chao1 richness and affected the beta-diversity of intestinal microbiota. The higher microbiota diversity is generally considered to associate with the health improvement in pigs (Ober et al., 2017). Vo et al. (2017) also suggested that increased microbiota diversity in the nursery pigs can efficiently reduce the risk of allergic diseases due to the function of microbiota in modulating the immune system. In addition, a previous study showed that increasing diet complexity by including more feedstuffs could be a sustainable method to increase the microbiota diversity of pigs (Fouhse et al., 2016). When all animal protein supplements were replaced by SPC, the reduction of diet complexity caused the reduction of microbiota diversity in pigs. The reduced diversity of mucosa-associated microbiota was a consequence of the decreased relative abundance of *Helicobacteraceae* and *Campylobacteraceae*. *Helicobacteraceae* and *Campylobacteraceae*, belonging to the phylum of Proteobacteria, are associated with unhealthy pigs and have been reported to cause the reduction of mucous layer protection and proliferative enteritis in pigs (Kamei et al., 2015; Zhang et al., 2017). This shift could associate with the changes in dietary protein supplements. When all animal protein supplements were replaced by SPC, it changed the physicochemical conditions and the substrate availability in the intestine of nursery pigs. Plant source protein can modulate the intestinal microbiota increasing the abundance of beneficial bacteria (Cao et al., 2016; Duarte and Kim, 2022). Rist et al. (2013) indicated that the intestinal microbiota of pigs was sensitive to the dietary protein source, and the highly digestible protein sources could reduce protein fermentation and proliferation of potentially pathogenic bacteria in the intestine.

Intestinal morphology is related to the nutrient digestion and absorption capacity of the intestine (Xiong et al., 2015). Studies have indicated that the antigens in soy proteins can negatively affect intestinal morphology, proliferative index, and relative enterocyte migration rate in the intestine of nursery pigs (Qin et al., 2002; Qiao et al., 2003). However, no difference in intestinal morphology was observed in jejunal tissue among treatments in this study. This result can be explained by the low concentration of antigens in SPC. Ma et al. (2019) reported that the use of 9% enzyme-treated SBM replacing 7.38% fish meal did not change the morphology of jejunum in weaned pigs. In addition, apparent ileal digestibility of nutrients was not affected by increasing SPC supplementation by replacing animal protein supplements. However, Yang et al. (2007) reported that SPC can improve the digestibility of nutrients compared to other soy protein supplements. Xie et al. (2016) also indicated that SPC can partly replace fish meals in the shrimp diet without negatively affecting the apparent digestibility of nutrients.

In conclusion, increasing SPC supplementation by replacing animal protein supplements in diets for nursery pigs reduced growth performance due to decreased feed intake, which might be related to increased PYY in the jejunal tissue. However, based on an exponential model, 10.5 and 16.8% of animal protein supplements can be replaced by SPC without affecting BW gain and feed intake of nursery pigs, respectively. The complete replacement of animal protein supplements by SPC supplementation modulated the composition of jejunal mucosa-associated microbiota by decreasing the relative abundance of *Helicobacteraceae* and *Campylobacteraceae*, whereas having no effect on intestinal immune status, intestinal oxidative stress status, intestinal morphology, and the AID of nutrients in nursery pigs.

## Abbreviations

AA: amino acid
ADFI: average daily feed intake
ADG: average daily gain
AID: apparent ileal digestibility
BW: body weight
CCK: cholecystokinin
CP: crude protein
DM: dry matter
DNA: deoxyribonucleic acid
EE: ether extract
ELISA: enzyme-linked immunoassay
GE: gross energy
GLP-1: glucagon-like peptide 1
G:F: gain to feed ratio
IL-8: interleukin-8
IgA: immunoglobulin A
IgG: immunoglobulin G
MDA: malondialdehyde
OTUs: operational taxonomic units
PBS: phosphate-buffered saline
PWD: post-weaning diarrhea
PYY: peptide tyrosine tyrosine
SBM: soybean meal
SID: standard ileal digestibility
SPC: soy protein concentrate
TiO_2_: titanium dioxide
TNF-α: tumor necrosis factor alpha
VH:CD: villus height to crypt depth
